# Adaptive model to support business process reengineering

**DOI:** 10.7717/peerj-cs.505

**Published:** 2021-04-29

**Authors:** Noha Ahmed Bayomy, Ayman E. Khedr, Laila A. Abd-Elmegid

**Affiliations:** 1 Information Systems Department, Faculty of Computers and Artificial Intelligence, Helwan University, Ain-Helwan, Cairo, Egypt; 2 Information Systems Department, Faculty of Computers and Information Technology, Future University in Egypt (FUE), New Cairo, Cairo, Egypt; 3 Information Systems Department, Faculty of Computers and Artificial Intelligence, Helwan University, Ain-Helwan, Cairo, Egypt

**Keywords:** Business Process Reengineering, Critical Success Factors, Data Mining

## Abstract

The one constant in the world is change. The changing dynamics of business environment enforces the organizations to re-design or reengineer their business processes. The main objective of such reengineering processes is to provide services or produce products with the possible lowest cost, shortest time, and best quality. Accordingly, Business Process Re-engineering (BPR) provides a roadmap of how to efficiently achieve the operational goals in terms of enhanced flexibility and productivity, reduced cost, and improved quality of service or product. In this article, we propose an efficient model for BPR. The model specifies where the breakdowns occur in BPR implementation, justifies why such breakdowns occur, and proposes techniques to prevent their occurrence again. The proposed model has been built based on two main sections. The first section focuses on integrating Critical Success Factors (CSFs) and the performance of business processes during the reengineering processes. Additionally, it implements the association rule mining technique to investigate the relationship between CSFs and different business processes. The second section aims to measure the performance of business processes (intended success of BPR) by process time, cycle time, quality and cost before and after reengineering processes. A case study of the Egyptian Tax Authority (ETA) is used to test the efficiency of the proposed model.

## Introduction

**Figure 1:**
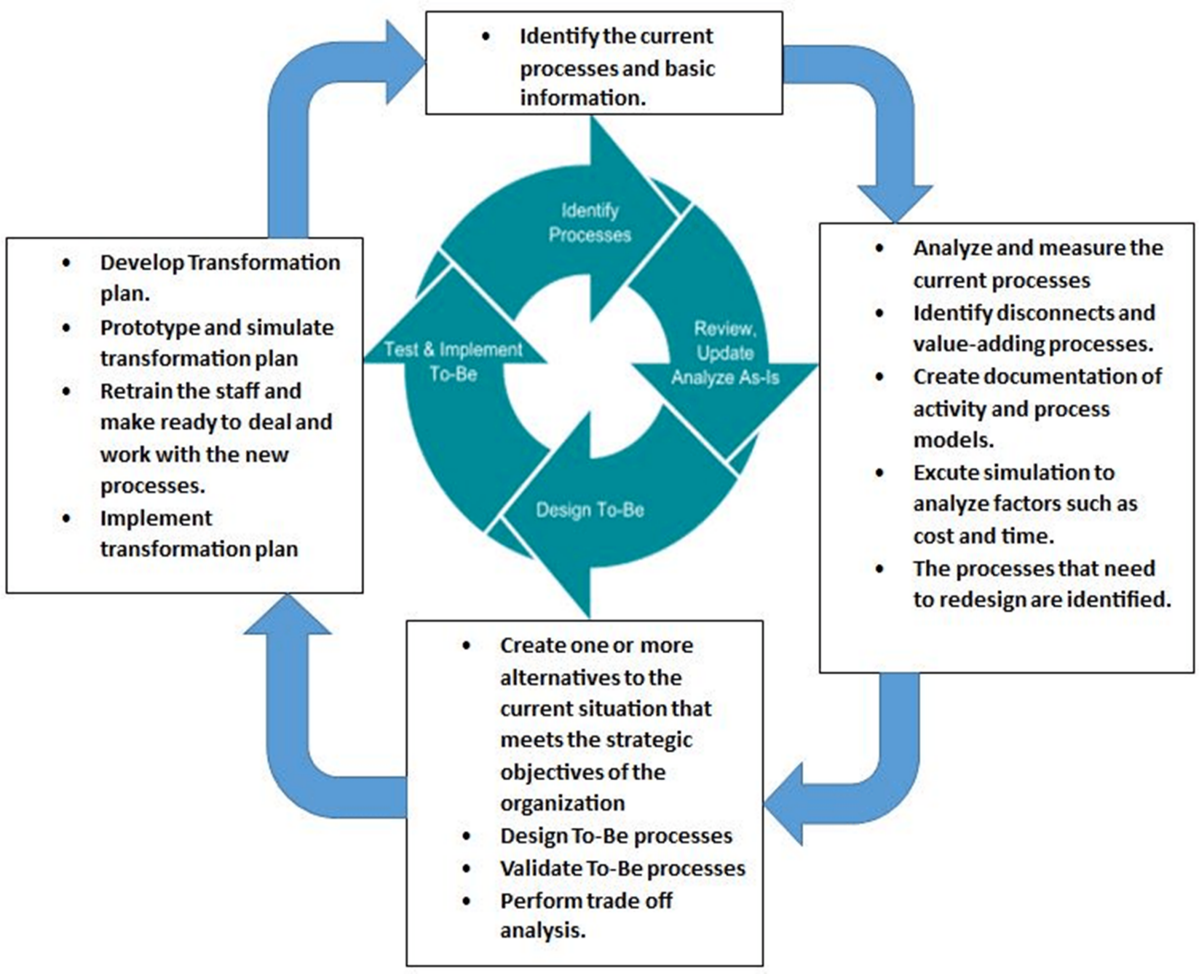
BPR life cyle (Khedr, Kholeif & Saad, 2017).

Re-engineering is the suggested solution when the current system doesn’t work satisfactorily or when critical system improvements are required to emulate competitive organizations. Elapatha & Jehan (2020) (p. 2), defined Business Process Re-engineering (BPR) as “the fundamental rethinking and radical redesign of business process to achieve dramatic improvements in critical, contemporary measures on performance”. Many organizations applied BPR since 1990 but the success rate was just 30%. The Business Process Re-engineering (BPR) cycle, shown in Fig. 1, consists of four steps that should be followed in order, where each step considers different aspects of re-engineering (Khedr, Kholeif & Saad, 2017).

Critical Success Factors (CSFs) are set of factors that should be identified and accomplished by the managerial level of an organization to increase the chances of successful implementation. The success of BPR implementation relies on identifying and analyzing critical success factors of BPR. CSFs can be categorized as internal (endogenous) and external (exogenous). The endogenous CSFs are related to the issues and conditions within the organization that the managers can control. The exogenous CSFs are related to the issues outside to the organization that the managers may not be able to control (Dubey & Bansal, 2013; Maleki & Beikkhakhian, 2011; Performance Measure Guide, 2019). In this research, the major CSFs of BPR are less bureaucratic (flatter structure), egalitarian culture and leadership, customer focus, change management, use of information technology, project management, adequate financial resources, collaborative working environment, top management commitment, and organizational structure.

For information about CSFs previously mentioned, refer to Cheng & Chiu (2008), Hussein et al. (2013), Iqbal, Nadeem & Zaheer (2015), Maleki & Beikkhakhian (2011), Mekonnen (2019) and Nzewi, Nzewi & Moneme (2015). From our point of view, organizations should adopt a Quality Management System (QMS) that is defined as a formal system that documents the processes, procedures, and responsibilities for achieving quality policies and objectives concept and application because it helps them achieve their goals and the need to work to create and provide the requirements for this concept (Stravinskiene & Serafinas, 2020).

Data mining is used to predict and extract patterns of full information. Association rule mining is one of data mining components. It is the most serious model that has been created and vastly studied by the data mining community (Khedr & Kok, 2006). Also, it uses association rules which are an important category of methods for discovering patterns in data. Association mining has been applied in many fields. The business field is considered one of the best areas in which association mining has been applied where finding purchasing patterns or connects between products is very useful for making decisions and efficient marketing (Khedr & Elseddawy, 2015). Examples of applications of association mining include discover patterns in biological databases, extraction of knowledge from software engineering metrics, web personalization, text mining, telecommunication networks, market and risk management, inventory control etc. The goal of association rule mining is to extract interesting correlations, frequent patterns and associations or casual structures among sets of items in the transaction databases or other data repositories. Slimani & Lazzez (2014) stated association rule mining first time is introduced by Agrawal, Imielinski & Swami (1993). Association rule (If-Then rule) is defined “it includes picking out the unknown interdependence of the data and finding out the rules between those items”. There are two measures with association rules which quantify the support and confidence of the rule for a given data set.

**Figure 2:**
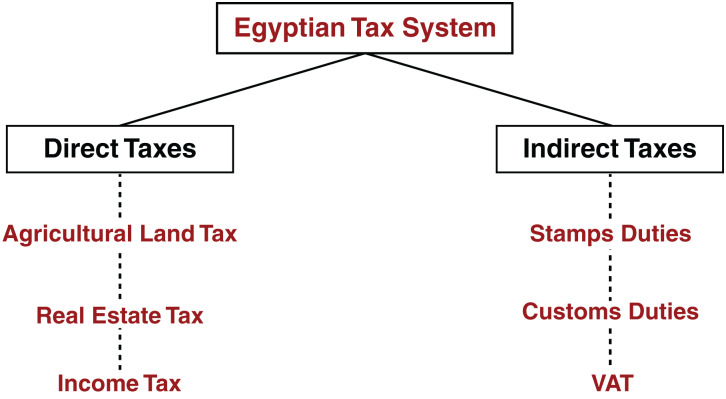
Structure of Egyptian tax system (Egypt Tax Summaries, 2018).

Taxes are the lifeblood that supplies government treasuries in countries with revenues and funds their civilized development projects. The tax is considered to be the oldest financial source for the state due to the large amount of money that it provides to the state’s public treasury. Its importance has increased with its increasing share in the public revenue structure. As well as the large role that taxes play in achieving the country’s political, financial, social, economic and development goals. The importance of the tax comes from being a basis for the development of societies and working to redistribute wealth and get in among segments of society and benefit various individuals through services and development projects. The tax is an amount of money that is imposed by the governmental authority so that the state can implement important matters for it. Tax is imposed in different countries of the world, the methods and mechanisms for collection are different for it. The current structure of taxes types in Egypt, as directed in Fig. 2, includes both direct taxes and indirect taxes (Egypt Tax Summaries, 2018). Firstly, direct taxes play a large role in the Egyptian economy and are imposed on personal or corporate income. They are classified into three forms of taxes involving income tax, real estate tax and agricultural land tax. Secondly, indirect taxes are taxes levied on goods and services. They are also classified into three forms of taxes including value added tax (VAT), customs duties, and stamp duties. For more information about mentioned forms of taxes types, refer to Ali et al. (2017).

**Figure 3:**
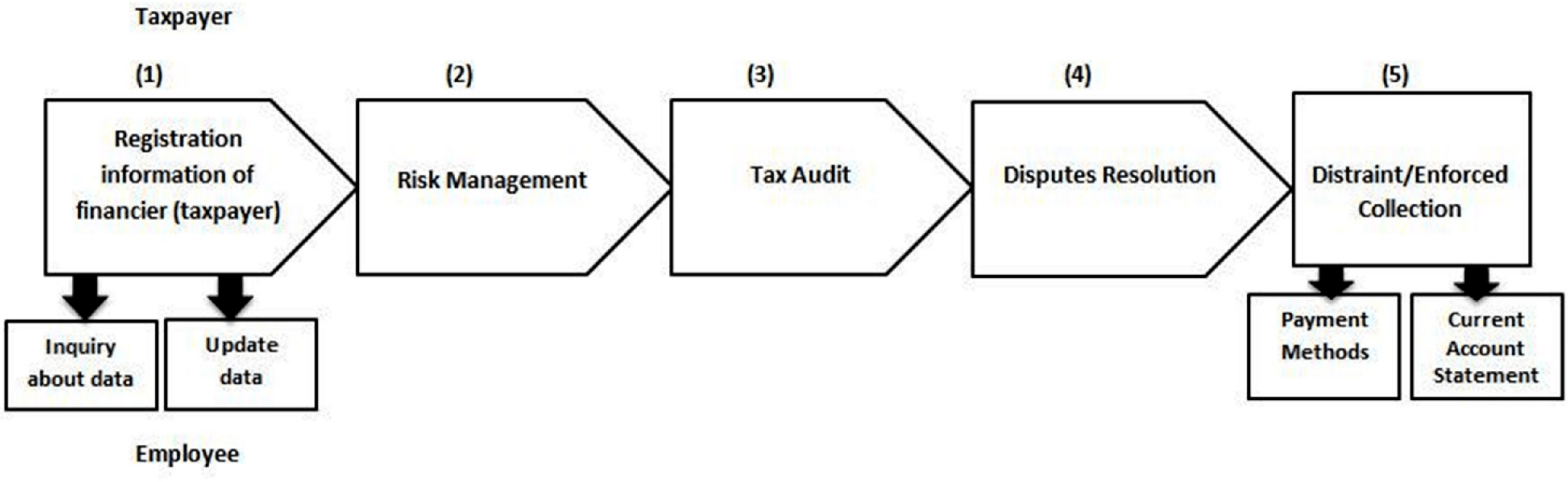
Core processes of tax in Egypt.

Each tax authority in Egypt consists of the same five main processes with four sub-processes that occur in sequential order and the stakeholders are the employee and the financier (taxpayer) that can be described in Fig. 3. This research focuses on income tax related to the personal tax (wages and salaries) in the case units for this study, which are used in the basic processes of taxes. Due to the confidentiality of the taxpayers’ data, the researcher’s request to obtain the data of the taxpayers or access their database was rejected to prepare a common database that includes the two types of taxes (direct and indirect) for the taxpayer to unify the tax return. Therefore, the researcher faced the difficulty in implementing To-Be registration process.

Customer Relationship Management (CRM) is described in the following aspects (Khedr, 2008): (1) CRM is a strategic focus on customer behavior and communication with customers; (2) CRM is a software technology for predicting the most valuable customer from collecting the customer data. (3) CRM is a business process mainly uses target customer data to improve the efficiency and effectiveness of the business. (4) CRM is a commitment to achieving customer satisfaction. From our point of view, Customer Relationship Management (CRM) is not for customers or goods, but also for taxpayers and public tax authority. To achieve the satisfaction of tax payers, the Egyptian Tax Authority must improve and deliver more superior service than expected. So, it is important to add CRM as a core business process with the key tax processes in Egyptian Tax Authority which will help to increase tax revenues in the state budget by building effective communication with financiers to better serve them, facilitate the collection of tax amount as soon as possible and achieving voluntary commitment that is part of the Egyptian Tax Authority’s vision. The main goal of CRM process is to resolve taxpayers’ complaints and maintain relationships with taxpayers.

This paper is organized into fourth sections as follows: “Literature Review” this section presents a literature review that has attempted to address business process reengineering models and comparison between these models and our proposed model. “Adaptive Model to Support Business Process Reengineering” this section describes building the proposed adaptive model to support business process reengineering that consists of two subsections A and B: (A) BPR Implementation and (B) BPR Success in order to follow the steps of its implementation. “Materials and Methods” this section clarifies the data collection methods and techniques. “Results and Discussion” this section describes step-by-step the implementation of the adaptive model of BPR in two units of Egyptian Tax Authority and discusses the findings. The end of paper provides conclusions of the overall results of the paper and recommendations for future work are proposed based upon the study findings.

## Literature Review

Awolusi & Onigbinde (2014), suggested a model that studies the relationship between CSFs of BPR and operational and organizational performances. Thus, examines the impacts of operational performance on organizational performance. By measuring operational performance that indicates to improve quality of products, cost reduction and high flexibility will give a positive effect on the organizational performance which relates to financial and non-financial measures to express the successful implementation of BPR. The effect of change of management system and culture, organizational structure, project planning and management and information technology infrastructure on the success of BPR (improving business performance) of all Nigerian oil and gas companies has been confirmed empirically excluding management support and competence.

Ayzatullova, Lyadova & Shalyaeva (2015), provided an approach to BPR during the integration of the Domain Specific Modeling (DSM) of business process and process mining tools. An analysis of the current approaches to business processes improvement and its restrictions have been showed. The process mining methods are connected to BPR stages and tasks. The advantages of using the domain specific modeling tools (DSM platforms, Language workbenches) have been confirmed.

They described a brief comparison of different visual languages notations and model transformation examples. Domain Specific Modeling (DSM) platform maintained mutual understanding between specialists. Meta Language DSM platform is the foundation of integration tools. Some domain-specific languages DSL (metamodels) and transformation have been presented. The complexity of analyst’s work is reduced by the implementation of integrated tools.

Iqbal, Nadeem & Zaheer (2015), presented a model that studies the relationship between critical success factors (CSFs) of BPR and Business Process Efficiency (PBE) that is defined as the levels of performance for business processes by reducing cost, cycle time, delays and duplications and Process Conflict (PC) that is defined as disputes and disagreements about resource delegation and job responsibilities, then examines the impacts of PBE and PC on organizational performance (financial and non-financial performances). Model is tested by using structural equation modeling Analysis of Moment Structures (AMOS). The findings clarify that CSFs has a positive effect on PBE. Thus, PBE will increase the performance. On the other hand, there is no relationship between CSFs and PC, so Process conflict will reduce the performance.

Omidia & Khoshtinata (2016), suggested a model that describes the effect of technical factors like (process management, change management) and organizational culture like (involvement, integration, capability) moderating the human factors on the implementation of BPR. Correlation tests and structural equation modeling were used for data analysis. Statistical Package for the Social Sciences (SPSS) and Analysis of Moment Structures (AMOS) software were used for data analysis. Accordingly, this model is needed for employees to recognize the company’s proven problems, the need to change, support the implementation of re-engineering and not be afraid of losing their jobs. Time and cost should be allocated for the re-engineering process and a training program for employees on new processes. Managers should encourage employees to participate in the design of new processes and motivate them so as not to resist the process change. The use of IT support is also essential in the implementation of reengineering in order to support decision-making and solve the challenges in the organization.

A framework for supporting redesign decision making is established by Phiphopsuthipaiboon & Boonsiri (2016). The framework is used to determine the necessary and unnecessary steps for the service processes in computer center that would be re-engineered through four phases of BPR life cycle. It focuses on reduce cycle time and steps in new process as a success of BPR. So, the step took a time over 1 hour called suspected unnecessary step.

According to the current conditions, all organizations need a basic and scientific model in order to help them achieve the desired results for a competitive advantage. Many studies popped up proposing models to find out the relationship between CSFs of BPR and the operational performance/the organizational performance, various others suggest methodologies and models to redesign business processes, each methodology had different phases and there is no assessment of the success of BPR. However, the scope to integrate CSFs and performance of business processes as a measure of BPR success during reengineering processes for a competitive advantage slightly disregarded by most of the researchers. Because critical success factors are an essential step in the process redesign stages, they must be studied to avoid the failure of BPR. On the other hand, most studies agreed on the study tools, as they were mostly the questionnaire, and sometimes they were based on personal interviews and the analysis of the questionnaire through using regression analysis and exploratory factor analysis to understand the causal relationship, with the exception of few of them mentioned above where data mining techniques were used. The following Table 1 is built to illustrate a comparison of the previous examples between BPR models and the proposed model.

**Table 1:** Comparison of BPR models.

Items	Awolusi & Onigbinde (2014)	Ayzatullova, Lyadova & Shalyaeva (2015)	Iqbal, Nadeem & Zaheer (2015)	Omidia & Khoshtinata (2016)	Phiphopsuthipaiboon & Boonsiri (2016)	Proposed Model
1-Integrating CSFs and Performance of Business Process.	✗	✗	✗	✗	✗	✓
2-Measuring Performance of Business Process before Reengineering	✗	✗	✗	✗	✗	✓
3-Measuring Performance of Business Process after Reengineering.	✗	✗	✗	✗	✗	✓
4-Using data mining Techniques	✗	✓	✗	✗	✗	✓

So, the previous issues are handled by the proposed model as will be illustrated in the following section.

## Adaptive Model to Support Business Process Reengineering

**Figure 4:**
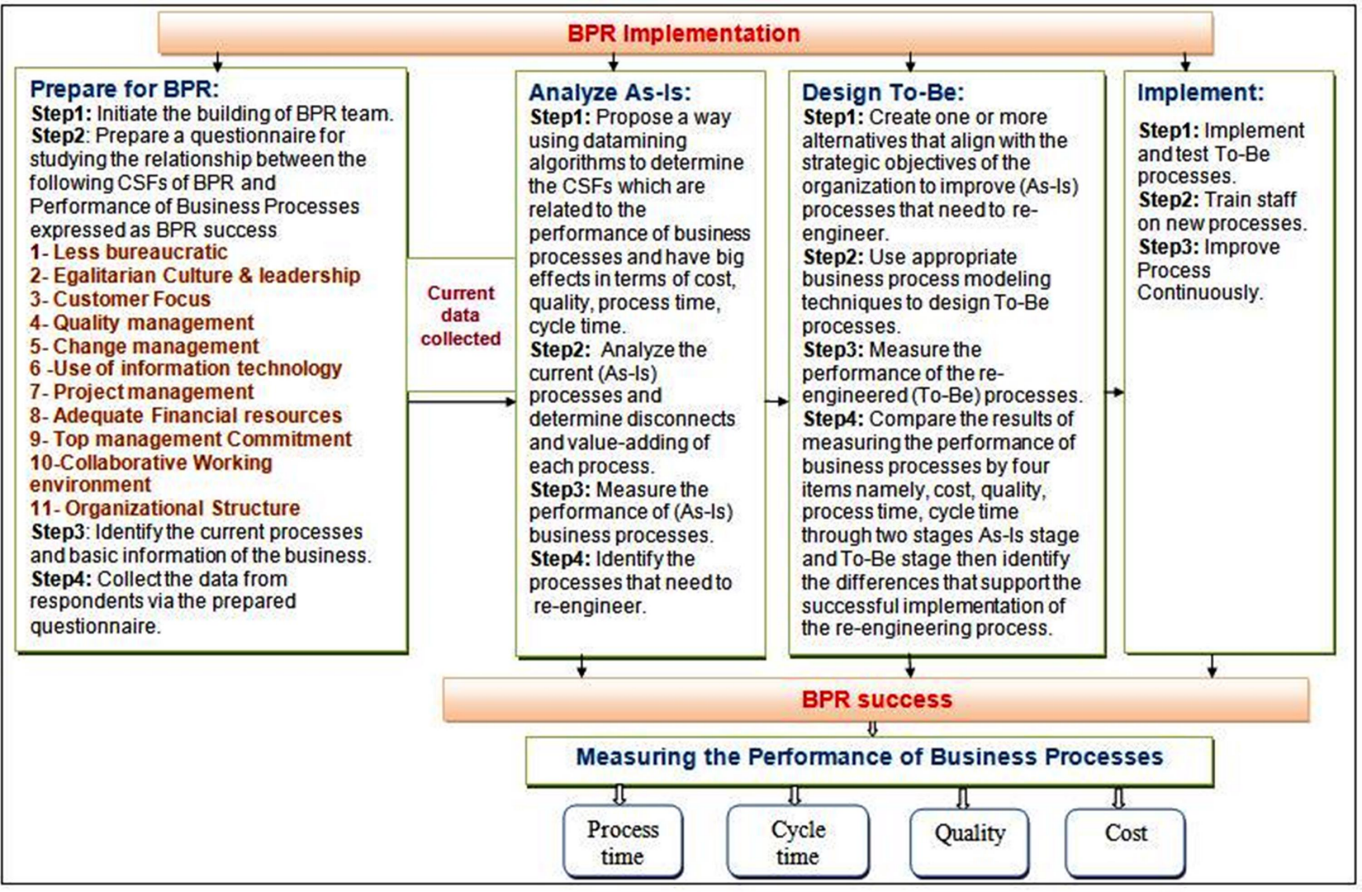
Adaptive model to support BPR.

Organizations require a scientific and basic model to meet their needs according to current circumstances. The main goal of Adaptive Model to Support Business process Reengineering is to specify where the breakdowns happen in BPR implementation, why they happen and how they can be prevented to guarantee successful implementation of reengineering business processes and improve its performance. The proposed model entitled Adaptive Model to Support BPR has been built based on BPR life cycle approach. There are number of factors that affect the BPR implementation are termed as CSFs of BPR. Upon the completion of BPR implementation, performance is measured before and after re-engineering by cost, time and quality (intended success of BPR). Due to the existing gaps in BPR researches, our model will focus in details on evaluating the effects of these CSFs on the performance of pre and post reengineered business processes. This is a model that is enclosed to its methodology underneath to implement the steps of model. According to Adaptive Model to Support BPR, it is implemented in two sections as shown in Fig. 4.

### A. Section 1: BPR implementation

The main goals of this section are to find and understand the associations between CSFs of BPR and the performance of business process by using apriori algorithm of association rule mining, identify and analyze the current business processes to detect disconnects and value-adding of each process as well as determine proposed changes to decide how to redesign some of the selected processes in order to help the organization achieve their business goals and strategies. BPR Implementation section is performed on four stages which are: (1) Prepare for BPR, (2) Analyze As-Is, (3) Design To-Be and (4) Implement. Each stage contains a series of sequential steps that are executed in order.

### B. Section 2: BPR success

The main goal of this section is to measure the performance of business processes through two stages which are Analyze As-Is for the existing business processes and Design To-Be for the re-engineering business processes in BPR Implementation section by cost, process time, cycle time and quality and compare the performance in these two stages to determine the variations expressed as BPR success. The main performance measures types are described as shown in Table 2 below.

**Table 2:** Main performance measures types.

Measure	Description
***Process time***	The necessary time for one or more inputs to convert into completed service or product through a given process. Business usually seeks to reduce the process time to a minimum of a particular good or service without compromising quality to the extent that consumers will buy less. According (Abdel-Fattah, 2015), Process time is expressed in three terms which customized in this study: *-Working time:* the amount of time which is indeed required for executing the task. *-Wait time:* the time which resources are free but have to stand still because of a lack of synchronization with other processes. *-Transfer time:* the time period in which the work item is moved, for example, the transfer of a piece of work or document, to another location including electronically.
***Cycle time***	The required time for one unit to pass through the process (Bhaskar, 2018). Total time from start to end of your process, as determined by you and your customer. The cycle time involves the process time, during which the unit is disposed to approximate the output, and the delay time, during which the business unit spends pending the next action.
***Quality***	ISO 9000 defines quality as the “degree to which a set of inherent characteristic fulfills requirements” (Brocke & Mending, 2018). There are various ways of looking at quality. Quality can be regarded as the perceived quality of a customer that is defined as external quality, or the perceived quality of the employee performing the task. This is called internal quality and indicates the state of work within the business process. Furthermore, There are differences between product quality and process quality. Quality is interlinked with other dimensions of are features, reliability, conformity, durability, ease of service and aesthetics for example, it is expressed in time. The produced or received percentage at the first time when meets criteria and specifications without any rework.
***Resource cost***	The assigned cost of the business based on the sum of the costs of the sub-activities that make up the process. This means the cost of the process that the customer orders it to produce a specific product from the beginning of the given process to its end (Dumas et al., 2018).

## Materials and Methods

The basic form of this research is descriptive and correlative. Descriptive research designs are used to gather information on current state of phenomena and describe the relationship between variables. Correlation research design is used to discover the relationship between two concepts. It is measured by a five—point scale ranging from 1 to 5, where “1 = strongly disagree, 2 = Disagree, 3 = neither disagree nor agree, 4 = Agree and 5 = strongly agree” (Jehan & Elapatha, 2020). Its most important features are simple to build and reliable, participants can read and complete it easily and more viable for measuring the good results. Ultimately, this case study is confirmed as a method for exploratory research. The main source of data collection for this research is surveys through the use of questionnaire. Survey research is appropriate way of generalizing from a sample to a population, allowing this effect to draw conclusions on the entire population. The population includes all managers, supervisors and employees of the Egyptian Tax Authority in two authorities (Joint Stock Companies and Helwan). The prepared questionnaire in this study divides into three parts as shown in Table 3. The first part A of the questionnaire, respondents clarifies background information about their name (optional), gender, education, job degree and how long they have worked in the organization. The second part B of the questionnaire consists of 33 statements about CSFs of BPR stemming from 11 factors adopted from (Cheng & Chiu, 2008; Iqbal, Nadeem & Zaheer, 2015; Maleki & Beikkhakhian, 2011; Mekonnen, 2019; Nzewi, Nzewi & Moneme, 2015; Sorunke & Nasir, 2016). The third part C of the questionnaire asks respondents to estimate the performance of the organization’s existing processes whence cost, time and quality. There were 1,123 questionnaire forms administered, of which 1,080 were usable, 19 forms were ignored due to missed vital data and 24 forms were received empty because respondents do not find enough time to fill out the questionnaire due to the large number of tasks assigned to them.

The Cronbach’s alpha was calculated to test the robustness of the data by the following formula adopted from Jehan & Alahakoon (2020).

$$\alpha =\frac{K}{K-1}\left(1-\frac{\sum_{i=1}^{K}{\sigma_{i}}^{2}}{{\sigma_{t}}^{2}}\right)$$

**Table 3:** List of variables and different cases.

Variable	Variable coding	Different cases		Cases coding
Background Information	Qa (No.question)	Name (Optional)		————
		Gender	Female	F
			Male	M
		Education	Less than Bachelor	LBD
			Bachelor	BD
			Higher than Bachelor	HBD
		Job Degree		————
		How long have you worked for this organization?	Less than five years	L.Five
			Five-Ten years	Five-Ten
			Ten for over	O.Ten
Critical Success Factors (CSFs) of Business Process Re-engineering (BPR)	Qb (No.question)	Strongly disagree		SD
		Disagree		D
		Neutral		N
		Agree		A
		Strongly Agree		SA
Performance of Business Process	Qc (No.question)	Strongly disagree		SD
		Disagree		D
		Neutral		N
		Agree		A
		Strongly Agree		SA

Here, K is the number of items in a scale, σ_***i***_^**2**^ is the variance of *i*th item and σ_**t**_^**2**^ is the variance of the scale (total) scores.

Awolusi & Onigbinde (2014) indicated that for acceptable reliability, the Cronbach’s alpha should be 0.7 and above based on the questions. All scales have reliability coefficients above 0.7 where Cronbach’ Alpha (CA) of 33 items of CSFs was 0.9582 and CA of three items of performance of business processes was 0.8543.

In all 1,080 (96.17% response rate) questionnaires were accepted and analyzed. The data analysis and processing relied on the application of data mining. Data mining is a statistical technique that used to analyze the results of the extracted data to provide knowledge in a humanized format for the visions of future development plans.

Waikato Environment for Knowledge Analysis (WEKA) is a free machine learning software that contains a set of algorithms for predictive modeling and data analysis. Association rule mining is used to detect the most frequent itemsets and connections between data items by apriori algorithm in WEKA tool which has some characteristics such as simplicity, easy implementation, and efficient algorithm for extracting all repeated itemsets (Ali & Hamed, 2018).

An Evaluation Framework for Business Process Modeling Techniques (BPMTs) is used to decide how to select appropriate model technique for Business Process (BP). This framework is divided into two phases which are the first phase of gathering requirements about the need of Business Process and the second phase of matching the needed requirements with the BPMTs characteristics. For more information about mentioned framework, refer to Abdel-Fattah, Khedr & Nagm Aldeen (2017).

## Results and Discussion

This section describes the step-by-step application of the model and its results as follows:

### Stage one: prepare for BPR:

This stage consists of four steps as follows:

#### Step 1: initiate the building of BPR team.

The BPR team is firstly created to prepare a pilot study and interviews with stakeholders to know how willing they are to accept the change in their business so as to avoid resisting the change when the implementation of the new changes is completed which can lead to the failure of the BPR and big loss of money and time. In addition to gathering all information about organization and its activities to analysis the overhead activities of the organization in order to determine which of them need to reengineer for keeping in touch with the real objectives of organization. At the end of the implementation of BPR, this team will train stakeholders on the new changes.

#### Step 2: prepare a questionnaire for studying the relationship between CSFs of BPR and performance of business processes expressed as BPR success.

The questionnaire form for studying the relationship between CSFs of BPR and Performance of Business Processes expressed as BPR success was constructed based on successful studies previously constructed in the related field of study. There are two independent variables; Critical Success Factors (CSFs) of Business Process Re-engineering (BPR) and performance of business process which used a 5-points scale.

#### Step 3: identify the current processes and basic information of the business.

The history of the tax sector in Egypt is described, the types of direct and indirect taxes, and the changes that have occurred in tax sector. This was followed by submission of selected two case units: Joint-Stock Companies Tax Authority and Helwan Tax Authority and determine the specialization and departments of each. The current processes (As-Is model) in Egyptian tax authority are illustrated in detail in terms of its goal, input, activities, output and limitations.

#### Step 4: collect the data from respondents via the prepared questionnaire.

The data collected on the number of employees in two case units. Based on results statistics after analyzing questionnaire form in terms of the Mean and Standard deviation, it has two main conclusions that can be illustrated:

The first conclusion is about the level of satisfaction among the tax employees with the current CSFs. The results show that most of employees are disagree with existing CSFs which activated in their business (all the means are below than 3 except 6) means that relate to managers constructively use the idea of their subordinates, Quality Management System (QMS) works successfully on decreasing errors, cost and pledging the identification and control of processes, organization seeks to empower staff and dismantle the current structure, train employees on the new process, develop strategic plans, employees are responsible for the results of their business and give employees the necessary validities to enable them to perform their tasks. The second conclusion is about the level of satisfaction among the tax employees with the current performance of business processes. The results show that in general most tax staff are disagree with their processes that performed with more time, cost and less quality. Limitations and obstacles exist in processes. Processes aren’t integrated (all the means are below 3).

### Stage two: analyze As-Is:

This stage consists of four steps as follows:

#### Step 1: propose a way using data mining algorithms to determine the CSFs which are related to the performance of business processes and have big effects in terms of cost, quality, process time, cycle time.

**Figure 5:**
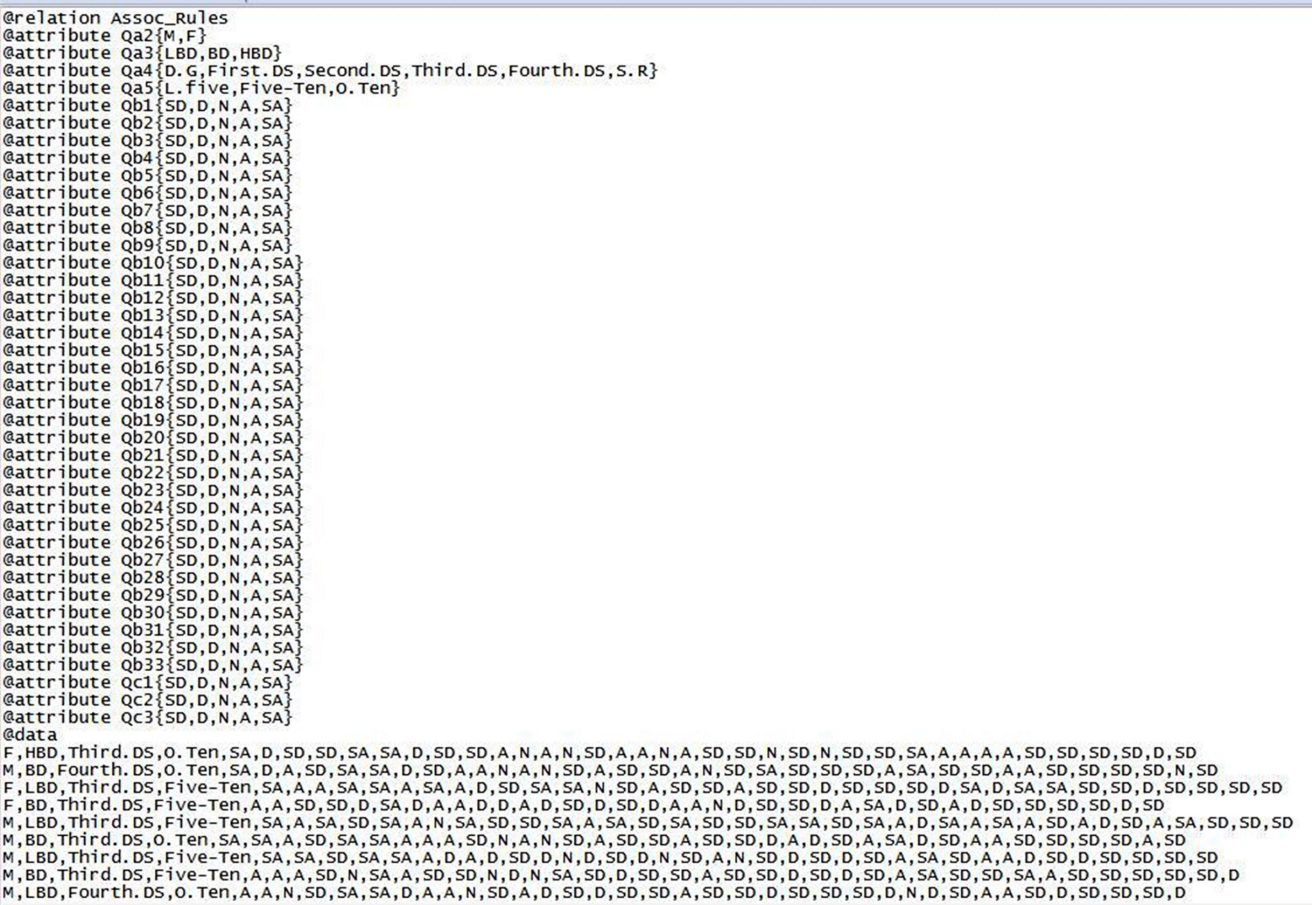
A sample of dataset in the ARFF.

**Figure 6:**
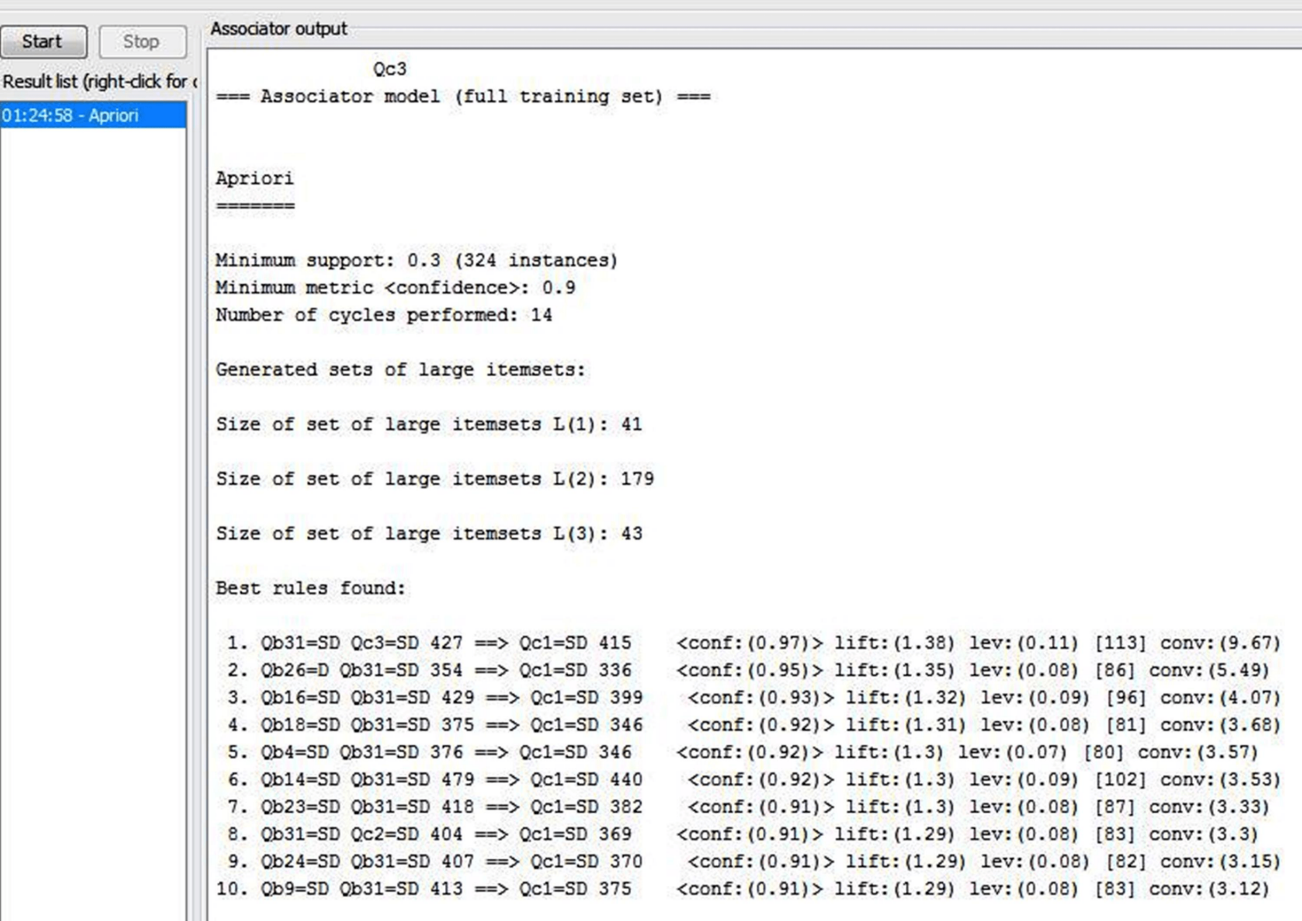
Discovered rules after applying apriori algorithm in WEKA.

1. Selecting the dataset which included 1,080 records with Comma Separated Values (CSV) format for tax employees’ responses in 2019 after eliminating 24 non-responding employees because of their large number of works, they do not have time to fill out the questionnaire form and 19 ignored for missing the vital variables by respondents.
2. Running Waikato environment for knowledge analysis (WEKA) tool, selecting the explorer application and loading dataset into WEKA tool. Before applying the datamining techniques, WEKA tool accepts determined format where the dataset transformed into Attribute-Relation File Format (ARFF) as shown in Fig. 5.
3. After loading and converting dataset, there are 40 attributes; Attribute of name Qa1 is ignored because it is not the focus of this study, in addition to being optional.
4. Applying the apriori algorithm for association rules discovery which achieves the relationship between Critical Success Factors (CSFs) of Business Process Reengineering (BPR) and Performance of Business Processes as shown in Fig. 6.
5. New rules are discovered through the apriori algorithm for association rules and examining the output to select the suitable required schema which enhance the performance of processes in joint stock companies tax authority and helwan tax authority (as sample units of Egyptian Tax Authority) as shown in Table 4.
6. Filtering new rules according to the suitable required schema: {Qb(—) Qb(—) == <Qc(—)} which help the Egyptian Tax Authority to determine the best CSFs that impacts on the performance of current processes for focusing on them to enhance the performance of BPs after implementing BPR as shown in Table 5. There are suitable eight associations rules after filtering new rules and two rules which are ignored because they don’t match with suitable required schema.
7. Testing the validity of the questionnaire’s answers according to the following Table 6 that shows our constraints by two parameters which are intuitively estimated by the users from the result of applying apriori algorithm. Depending on the choice of those thresholds, association rule mining algorithms can generate best rules.
8. The support of an association rule given by S(X⇒Y) is the proportion of the number of questionnaire answers including both X and Y (|T_X_∩|T_Y_|) to the total number questionnaire answers of |D|. S(X⇒Y) is given by the following formula (Slimani & Lazzez, 2014). The association rules related to questionnaire answers are given in the following Table 7 (All rules have a support higher than 30%).

$$S(X\Rightarrow Y)=\frac{|T_{X}\cap |T_{Y}|}{|D|}$$

**Table 4:** Association rules found by the WEKA data mining technique through apriori algorithm for the questionnaire of CSFs and performance of BP.

Serial	Association rules findings for the relative questions	
1	Qb31=SD Qc3=SD 427==> Qc1=SD 415	C = 97%
2	Qb26=D Qb31=SD 354 ==> Qc1=SD 336	C = 95%
3	Qb16=SD Qb31=SD 429 ==> Qc1=SD 399	C = 93%
4	Qb18=SD Qb31=SD 375 ==> Qc1=SD 346	C = 92%
5	Qb4=SD Qb31=SD 376 ==> Qc1=SD 346	C = 92%
6	Qb14=SD Qb31=SD 479 ==> Qc1=SD 440	C = 92%
7	Qb23=SD Qb31=SD 418 ==> Qc1=SD 382	C = 91%
8	Qb31=SD Qc2=SD 404 ==> Qc1=SD 369	C = 91%
9	Qb24=SD Qb31=SD 407 ==> Qc1=SD 370	C = 91%
10	Qb9=SD Qb31=SD 413 ==> Qc1=SD 375	C = 91%

**Table 5:** Suitable association rules from the new rules discovered after applying apriori algorithm.

Serial	Suitable association rules after filtering new rules	
1	Qb26=D Qb31=SD 354 ==> Qc1=SD 336	C = 95%
2	Qb16=SD Qb31=SD 429 ==> Qc1=SD 399	C = 93%
3	Qb18=SD Qb31=SD 375 ==> Qc1=SD 346	C = 92%
4	Qb4=SD Qb31=SD 376 ==> Qc1=SD 346	C = 92%
5	Qb14=SD Qb31=SD 479 ==> Qc1=SD 440	C = 92%
6	Qb23=SD Qb31=SD 418 ==> Qc1=SD 382	C = 91%
7	Qb24=SD Qb31=SD 407 ==> Qc1=SD 370	C = 91%
8	Qb9=SD Qb31=SD 413 ==> Qc1=SD 375	C = 91%

**Table 6:** Thresholds of support and confidence.

Parameters	Itemsets basis (%)
Minimum support (S)	30
Minimum confidence (C)	90

**Table 7:** Testing the validity of the questionnaire answers.

Serial	Suitable associations rules		
1	Qb26=D Qb31=SD 354 ==> Qc1=SD 336	S = 31%	C = 95%
2	Qb16=SD Qb31=SD 429 ==> Qc1=SD 399	S = 37%	C = 93%
3	Qb18=SD Qb31=SD 375 ==> Qc1=SD 346	S = 32%	C = 92%
4	Qb4=SD Qb31=SD 376 ==> Qc1=SD 346	S = 32%	C = 92%
5	Qb14=SD Qb31=SD 479 ==> Qc1=SD 440	S = 40%	C = 92%
6	Qb23=SD Qb31=SD 418 ==> Qc1=SD 382	S = 35%	C = 91%
7	Qb24=SD Qb31=SD 407 ==> Qc1=SD 370	S = 34%	C = 91%
8	Qb9=SD Qb31=SD 413 ==> Qc1=SD 375	S = 34%	C = 91%

#### Step 2: analyze the current (As-Is) processes and determine disconnects and value-adding of each process.

The current processes (As-Is models) of taxes are illustrated in detail. Analysis the current processes in Egyptian Tax Authority is very important, because they are previously modeled manually without using a specific modeling technique. So, it is important to model the current processes for analysis that will help in finding out the problem of detecting errors in the model such as redundancy, ambiguity etc.

#### Step 3: measure the performance of (As-Is) business processes.

In Joint Stock Companies Authority (for 122 sample of soft copy tax returns and 236 hard copy tax return), total of cycle time is 563 h, total of quality is 66% and total of cost for all processes is 7,756,250 EGP while in Helwan Authority (172 samples of hard copy tax returns), total of cycle time is 575 h, total of quality is 52% and total of cost for all processes is 7,655,250 EGP.

#### Step 4: identify the processes that need to re-engineer.

BPR team will decide which business processes that need to reengineer based on analysis the gathered information of two authorities and its activities in addition to measuring the performance of current (As-Is) business processes of each authority as presented in previous two steps in this stage. BPR team finds that all processes need to reengineer.

### Stage three: design To-Be:

This stage consists of four steps as follows:

#### Step 1: create one or more alternatives that align with the strategic objectives of the organization to improve (As-Is) processes that need to re-engineer.

The most important critical success factors are discussed that need to be reengineered in Egyptian Tax Authority which align with its strategic objectives and impact on the current processes as follows:

*Critical success factors of BPR findings from association rules:*

**1. Organizational structure**

The organizational structure is the key factor of success factors that has a significant impact on re- engineering the processes in Joint Stock Companies tax authority and Helwan tax authority. The structure of each organization consists of different aspects such as values, traditions, philosophies, hierarchies, authorities and rules (Jurisch et al., 2012).

The Egyptian Tax Authority is striving to adopt a unified organizational structure for direct and indirect taxes based on the Integration Law in 2006. From our point of view, this caused that the Egyptian Tax Authority needs bureaucratic models, which require the distribution of responsibilities among the different departments and other organizations as well as adjusts its procedures to suit the integration of the two types of taxes. It is necessary to link all databases related to the affiliated authorities of the Egyptian Tax Authority or the authorities that the Egyptian Tax Authority needs to share data with it, such as the civil registry in order to ensure the accuracy of the taxpayer’s data and the Ministry of Finance. This facilitates the taxpayer’s submission of one tax return that includes all taxes that must be collected from him to combat tax evasion based on building a unified organizational structure. This confirms the BPR principle that states “treat geographically dispersed resources as though they were centralized”. Some respondents indicated during the interview that the authority needs to establish a risks and crises management unit that works to solve the crisis, anticipate crisis before it occurs and work to avoid it. Effective communication channels must be established among all departments of the authority.

***2. Use of information technology***

The initiative of the Egyptian Tax Authority to change old processes using information technology is not BPR, but it is automation. There is a misunderstanding in BPR initiatives that give a lot of focus to information technology in the redesign processes that are not among BPR principles. Information technology must be treated as an executor for desirable reengineered processes.

So far, the tax processes in Egypt are done manually although the electronic payment process has already been developed to some extent and the taxpayers have some electronic options to pay off their liabilities. However, it needs further analysis to enable taxpayers to easier and safer to pay tax liabilities to the Egyptian Tax Authority. To implement the changes to the processes to be re-designed using information technology must take into account the following:

- All information systems within the authority must be effectively integrated and tax procedures checked to formulate them in line with the new implementation of processes, so that duplication of responsibilities can be averted and easily removing non-value-added transactions from the systems.
- The effectiveness of information technology infrastructure must be measured quantitatively to ensure its success.
- Proper use of all information technology related resources including desktop computers, laptop computers, servers, network equipment, printers, scanners, mobile Internet connection devices, software etc. that invest in BPR to implement reengineering of taxes processes at the expected level of quality.

***3. Adequate financial resources***

BPR is very expensive process. Respondents indicated that top management does not allocate capital or make plans to finance the re-engineering of existing processes. And that it is necessary to provide all the requirements and work tools to help the Authority’s employees to finish the work assigned to them in an easy way.

It is necessary for the Egyptian Tax Authority to draw up a budget plan to re-engineer the current processes, as it is a long-term investment for the Authority and will benefit from it with a lot of benefit. The capital allocated to the required changes in the Authority should not affect its obligations, workers’ wages, or any costs to its customers. We recommend that a training plan is prepared, as part of the financing plans for the BPR process, by the top management in the authority for its employees to encourage them to accept new changes and to develop themselves.

***4. Egalitarian culture and leadership***

Based on what respondents indicated in the interview and the questionnaire, there is no communication between employees in the authority and managers or an exchange of views and opinions. They explain that their proposals need to be taken into account in carrying out administrative processes. The features of the innovative organizational culture and leadership are the effective use of employee ideas for the authority to achieve the desired results, coordination, employee participation and friendly interactions. We propose to hold a monthly meeting between the managers and employees in the Authority in order to discuss the proposals submitted distribute the tasks to each individual and set an executive plan to perform the tasks in a specific time.

**5. Change management**

Change management is a practice that the authority follows in aligning changes in organizational activities in order to meet challenges and meet customer needs. A best practice is the reward system which was one of the most important items that respondents mentioned with dissatisfaction and that the current system of rewards is completely unfair and needs to be reviewed.

Through the interview with the respondents, they indicated that it is important to study a new tax law that is in line with the current era, as the current law is marred by many difficulties, and many amendments have occurred and it does not meet the needs of the present time.

Also, all employees of the Authority need to retrain; each of them within the limits required by the work carried out by him due to the current training in the authority is insufficient and characterized by incompetence and inadequacy. Finally, because of BPR changes organizational processes, employees must have sufficient skills to carry out new tasks. Through an appropriate training program, employees will have an in-depth understanding of their new tasks.

***6. Customer focus***

There are three questions under this factor, namely organization provides training for employees to determine the interaction with customers for creating long-term relations with them, employees have the motivation to carry out an effective training and the organization is able to provide customers’ demands based on the effective analysis of their requests. These questions illustrate that BPR aims to build long- term relationships by providing the necessary training for employees to deal with taxpayers and paying the employee to obtain training in relation to his work, redesigning workflows with the aim of improving the efficiency and effectiveness of customer relationship which is a process suggested in this study in order to provide financers’ demands and solve their complaints.

**7. Top management commitment**

Top management must have sufficient knowledge of BPR implementation and make important decisions in the BPR implementation process. Top management must motivate staff and friendly interaction with the BPR team. Additionally, Top management should use information and communication technology (ICT) to enhance efficiency of public service delivery (Alahakoon & Jehan, 2020). Respondents indicated through the questionnaire, a great disagreement with the strategic plans laid down by the top management in the authority, and they need to develop to suit the requirements of taxpayers and attract them to the authority by adding new activities that take into account the quality improvement, reduce crises and reduce the percentage of errors.

Some CSFs were included in this research such as less bureaucratic, project management, quality management and collaborative working environment for which WEKA did not find a significant association due to data set's attributes of success factors differ in each industry or organization from the other, depending on its business strategy and the logistical services it provides.

#### Step 2: use appropriate business process modeling techniques to design To-Be processes.

To-Be Processes are designed with the most suitable modeling techniques that are provided in each As-Is process. Business Process Modelling Notation (BPMN) Modeling Technique is applied in To-Be processes in registration process, risk management process, tax audit process, distraint/ enforced collection processes and customer relationship management process, while Unified Modeling Language Activity Diagram (UML-AD) Modeling Technique is applied in disputes resolution process.

#### Step 3: measure the performance of the re-engineered (To-Be) processes.

In Joint Stock Companies Authority (for 408 electronic tax return), total of cycle time is 264.55 h, total of quality is 6% and total of cost for all processes is 4,861,750 EGP while in Helwan Authority (124 electronic tax return), total of cycle time is 231.30 h, total of quality is 6% and total of cost for all processes is 4,840,250 EGP.

#### Step 4: compare the results of measuring the performance of business processes by four items namely, cost, quality, process time, cycle time through two stages As-Is stage and To-Be stage then identify the differences that support the successful implementation of the re-engineering process.

Findings comparison and discussion:

The performance of business processes has been measured by four items namely, cost, quality, process time and cycle time through two stages As-Is stage and To-Be stage then identifies the differences that support the successful implementation of the re-engineering process. BPR team decided to focus on eliminating unnecessary costs by reducing the cost of pool for each activity, reducing the frequency of activities to improve the quality of processes and saving time through the re-engineering process as shown in the Tables 8 and 9 below. In Joint Stock Companies, total cycle time in As-Is is 563 h with six official working hours equivalent of 94 working days which leads to the need for additional hours to work in the quarterly period while total cycle time in To-Be is 264.55 h/quarterly equivalent of 44 working days in the rate of change 53%. The rate of change in quality of processes is 90% for five processes while the rate of change in total of resource cost is 37%. In Helwan Authority, total cycle time in As-Is is 575 h with six official working hours equivalent of 85 working days which leads to the need for additional hours to work in the quarterly period while total cycle time in To-Be is 231.30 h/quarterly equivalent of 39 working days in the rate of change 53%. The rate of change in quality of processes is 88% for five processes instead of four processes as in As-Is while the rate of change in total of resource cost is 37% like in the other authority.

**Table 8:** Comparison of measuring the performance of business processes in stage analyze As-Is and stage design To-Be at joint stock companies authority.

Process time	As-Is	To-Be	Difference	% Change
Transfer time (hours)	60 h/quarterly	1.40 h/quarterly	58.20	97%
Wait time (hours)	95 h/quarterly	39.15 h/quarterly	55.45	58%
Working time (hours)	360 h/quarterly	224 h/quarter	136 h/per quarterly	38%
Cycle time				
Cycle time (hours)	563 h/quarterly	264.55 h/ quarterly	298.5 hour/ quarterly	53%
Quality				
Frequently occurring %	66%	6%	60%	90%
Total number of processes	5	5	0	0
Resource costs				
Paper works costs (including papers of Imaging and Printing, templates)	MIN.:187,500 per quarterly	22,500 per quarterly	165,000	88%
Necessary materials costs (files for archiving, CD, Calculators and Pens etc.)	Min.:150,000 per quarterly	17,750 per quarterly	132,250	88%
Purchase/maintenance costs of computers, printers, photocopiers, routers and switches.	MIN.:217,500 per quarterly	22,250 per quarterly	195,250	90%
Administrative Wages and Salaries	6,675,000 per quarterly	4,675,000 per quarterly	2,000,000	30%
***Total Varied Costs***	***7,230,000***	***4,737,500***	***2,492,500***	***34%***
Maintenance costs for administrative buildings	75,000 per quarterly	75,000 per quarterly	0	0%
***Total Fixed Costs***	***75,000***	***75,000***	***0***	***0%***
Office Equipment Depreciation	73,750 per quarterly	12,500	61,250	83%
Administrative Building Depreciation	60,000 per quarterly	18,250	41,750	70%
Incidental Expenses Cost	317,500	13,500	304,000	96%
***Total of running Cost***	***451,250***	***49,250***	***402,000***	***89%***
**TOTAL COSTS**	**7,756,250**	**4,861,750**	**2,894,500**	**37%**

**Table 9:** Comparison of measuring the performance of business processes in stage analyze As-Is and stage design To-Be at Helwan authority.

	As-Is	To-Be	Difference	% Change
Process time				
Transfer time (hours)	102 h/quarterly	1.20 h/quarterly	100.40 h/quarterly	98%
Wait time (hours)	95 h/quarterly	35.10 h/quarterly	59.50 h/quarterly	62%
Working time (hours)	352 h/quarterly	195 h/quarterly	157 h/quarterly	45%
Cycle time				
Cycle time (hours)	575 h/quarterly	231.30 h/quarterly	343.30 h/quarterly	60%
Quality				
Frequently occurring %	52%	6%	46%	88%
Total number of processes	4	5	1	25%
Resource costs				
Paper works costs (including papers, Imaging and Printing)	MIN.:160,000 per quarterly	77,000 per quarterly	19,250	88%
Necessary materials costs (Files for archiving, CD, Calculators and Pens etc.)	Min.:107,500 per quarterly	12,750 per quarterly	94,750	88%
Purchase / maintenance costs of computers, printers, photocopiers, routers and switches.	MIN.:132,500per quarterly	12,500 per quarterly	120,000	91%
Administrative Wages and Salaries	5,605,750 per quarterly	2,949,750 per quarterly	2,656,000	47%
***Total Varied Costs***	***7,230,000***	***4,737,500***	***2,492,500***	***34%***
Maintenance costs for administrative buildings	75,000 per quarterly	75,000 per quarterly	0	0%
***Total Fixed Costs***	***75,000***	***75,000***	***0***	***0%***
Office Equipment Depreciation	51,500 per quarterly	6,750	44,750	87%
Administrative Building Depreciation	54,250 per quarterly	13,000	41,250	76%
Incidental Expenses Cost	244,500	8,000	236,500	97%
***Total of running Cost***	***350,250***	***27,750***	***322,500***	***92%***
**TOTAL COSTS**	**7,655,250**	**4,840,250**	**2,815,000**	**37%**

There are significant differences in time, cost, and quality based on previous comparisons to measure the performance of processes through interviewing the respondents of employees, supervisors and top management for introducing sufficient and relevant arguments to our case study. Respondents have pointed out that there are several reasons that led to these differences, which are the obstacles in the current processes (As-Is), and they are as follows: (1) repeating processes due to the lack of a unified system, (2) processes are executed manually and not electronically due to lack of readiness for technical change, (3) lack of a working environment commensurate with the tasks assigned to employees to reduce the percentage of errors, (4) the absence of communication channels between the departments of the Authority or between the employees and the top management to take their proposals into consideration in implementing the processes. (5) the lack of well-studied strategic plans to amend the organizational structure or tax law that is compatible with the current era. (6) activating the risk management process with all authorities instead of the audit process employees performing both processes that need high efficiency and accuracy to detect the risks and work to avoid it. All these reasons are due to the failure to study the factors that affect the processes that are previously explained above and applied in each process assigned to them in the new system (To-Be) to become factors of success rather than reasons/factors for the failure of processes in the authority for improving the performance.

### Stage four: implement:

This is the final stage of the model that consists of the following three steps:

#### Step 1: implement and test To-Be processes.

To-Be processes are implemented in all units of ETA. Then, we should continuously test process performance (cost, time and quality) to make improvements on them in addition to create an executive plan based on performed BPR to ensure that improved processes are activated over a long-term period.

#### Step 2: train staff on new processes.

After the completion of the new processes implementation, all employees of the Authority must be trained on them before embarking on their actual work in the Authority and generalize them so that these processes are applied by the employees properly and also give the desired results from their implementation.

#### Step 3: improve process continuously.

Action progress is accomplished by conducting questionnaires and conversations with those who did not initially participate directly with the re-engineering to know how much more informed the people feel, how much more commitment the top management presents and how well the BPR teams are agreed in the wider perspective of the authority. Total Quality Management (TQM) is used as a tool to handle with different problems encountered throughout the BPR effort and to continually enhance the process.

## Conclusions

The model is used to determine where the breakdowns happen in BPR implementation, why they happen and how they can be prevented. This research paves the way for integrating CSFs of BPR and the performance of business processes in order to enhance processes and support successful implementation of BPR. According to the results, there are seven identified factors impacting on BPR success (as expressed by measuring performance of business processes) by using data mining techniques includes: (1) organizational structure, (2) use of information technology, (3) adequate financial resources, (4) egalitarian culture and leadership, (5) change management, (6) customer focus and (7) top management commitment. These factors have been integrated with performance of business processes to implement reengineering the processes of Egyptian Tax Authority successfully through stages of proposed model. The performance of business processes has measured before and after reengineering processes in addition to determining the variation in performance that confirm the success of BPR through applying the adaptive model. There is a similarity in the results of improvement in both authorities through rate of change in time, quality, and resource cost. The improved performance of the processes leads to the Egyptian Tax Authority getting a good reputation and a good name. Taxpayers get the best services that facilitate the way they pay for the tax amount imposed on them. New processes help tax employees to perform their tasks with great accuracy and high efficiency to reduce taxpayer complaints and avoid tax evasion.

We have attempted to build a proposed model that fits all industries but it has been applied to one industry such as taxes that is appropriate for our data set and this model extracted the factors affecting on this industry. It lacks some development and updating to be fit other industries because each one is different from other. Therefore, we recommend that we update the model to be applicable with different data set of other industries through doing globalization in order to extract many different critical success factors that influencing on performance of business processes then we can get a standard model for all industries.

On account of time limitations, some substantial issues have been left outside the scope of this study. Therefore, we recommend that it is necessary to measure customer satisfaction with the service provided to them after reengineering processes in a pilot test of processes. Besides, the benefits of applying business process reengineering (BPR) should be identified via our proposed model on organizational performance. The other direction of future work aims at giving more concentration on the Business Process Reengineering software tools which let the performance of business process to be estimated in order to support business process reengineering and making more implementation on the proposed model of BPR.

## Supplemental Information

10.7717/peerj-cs.505/supp-1 Supplemental Information 1 Association Rules file.csv.

## Additional Information and Declarations

### Data Availability

The following information was supplied regarding data availability: The dataset with 1080 records in Comma Separated Values (CSV) format of tax employees’ responses in 2019 is available in the Supplemental File. Analysis was conducted by: 1. Running Waikato environment for knowledge analysis (WEKA) tool, selecting the explorer application and loading dataset into WEKA tool. 2. Loading and converting dataset into Attribute-Relation File Format (ARFF). 3. Applying the Apriori algorithm for association rules discovery which achieves the relationship between Critical Success Factors (CSFs) of Business Process Reengineering (BPR) and Performance of Business Processes.
